# Safety, tolerability, and efficacy of high versus low-dose, short versus long-course daily primaquine for the radical cure of uncomplicated *Plasmodium vivax* malaria in children under 15 years of age: an open-label, non-inferiority, randomized controlled trial (CHILDPRIM)

**DOI:** 10.1186/s12936-025-05686-y

**Published:** 2025-12-22

**Authors:** Ana Luisa O. Pacheco, Aretha G. Omena, Djane C. Baía-da-Silva, Tyane A. P. Jardim, Debora C. B. Silva, Adriana P. B. Lopes, Laila R. A. Barbosa, Ingrid G. Souza, Renata F. Araujo, Luis O. S. Nogueira, Suianne C. N. Vale, Gisely C. Melo, Jady S. M. Cordeiro, Quique Bassat, Vanderson S. Sampaio, Valéria D. S. Lima, Flor E. Martinez-Espinosa, Maria Paula G. Mourão, Wuelton M. Monteiro, Maria Graças C. Alecrim, Jose Diego Brito-Sousa, Marcus V. G. Lacerda

**Affiliations:** 1 Fundação de Medicina Tropical Dr Heitor Vieira Dourado, Manaus, Brazil; 2 Universidade do Estado do Amazonas, Manaus, Brazil; 3 Instituto Leônidas & Maria Deane, Fiocruz, Manaus, Brazil; 4 Universidade Nilton Lins, Manaus, Brazil; 5 Universidade Federal do Acre, Cruzeiro Do Sul, Brazil; 6 ISGlobal, Barcelona, Spain; 7 Centro de Investigação Em Saúde de Manhiça (CISM), Maputo, Mozambique; 8 ICREA, Pg. Lluís Companys 23, 08010 Barcelona, Spain; 9 Institut Clínic de Medicina I Dermatologia, Hospital Clínic de Barcelona, Barcelona, Spain; 10 Facultat de Medicina I Ciències de La Salut, Universitat de Barcelona (UB), Barcelona, Spain; 11 Pediatrics Department, Hospital Sant Joan de Déu, Universitat de Barcelona, Esplugues, Barcelona, Spain; 12 CIBER de Epidemiología y Salud Pública, Instituto de Salud Carlos III, Madrid, Spain; 13 Secretaria Municipal de Saúde, Cruzeiro do Sul, Brazil; 14 Duke Global Health Institute, Duke University, Durham, USA; 15 University of Texas Medical Branch, Galveston, USA

**Keywords:** *Plasmodium vivax*, Malaria, Children, Paediatric, Primaquine, 8-Aminoquinolines, Relapse, Recurrence

## Abstract

**Background:**

Primaquine (PQ) is widely used to prevent *Plasmodium vivax* relapses. However, the most efficacious and safest dose is unknown, particularly in children. This trial assessed the safety, tolerability, and efficacy of two high-dose PQ regimens compared with standard of care (SoC) in children with *P. vivax* infections in the Brazilian Amazon.

**Methods:**

CHILDPRIM was an open-label, randomized clinical trial conducted in Manaus and Cruzeiro do Sul, Brazilian Amazon, from August 2021 to January 2025. The study evaluated the non-inferiority of high-dose PQ regimens in terms of safety, tolerability, and parasitological response at day 180 compared to the low-dose regimen in children under 15 years of age with uncomplicated *P. vivax* malaria. Participants were randomized (1:1:1) to receive: (1) Brazilian routine standard-dose PQ (3.5 mg/kg over 7 days)–0.5 mg/kg/day; (2) high-dose PQ long-course (7.0 mg/kg over 14 days)–0.5 mg/kg/day; or (3) high-dose PQ short-course (7.0 mg/kg over 7 days)–1.0 mg/kg/day, after glucose-6-phosphate dehydrogenase (G6PD) deficiency screening using the quantitative SD Biosensor. All participants were followed for 180 days. The primary outcomes were the proportion of participants experiencing adverse events of any intensity and the proportion of failures up to day 180 between groups.

**Results:**

A total of 100 individuals were randomized: 32 in the PQ 3.5 mg/kg over 7d arm, 34 in the PQ 7.0 mg/kg over 14d arm, and 34 in the PQ 7.0 mg/kg over 7d arm. The most common adverse events were methaemoglobinaemia, anaemia, and gastrointestinal symptoms. Higher doses of PQ resulted in more adverse events, but no more serious adverse events. Participants in the PQ 3.5 mg/kg over 7d arm presented a higher risk of recurrence at 42 and 180 days, which is why the trial was halted after the second interim analysis. Kaplan–Meier estimates of the percentage of participants who were free from recurrence at day 180 were 50% in PQ 3.5 mg/kg over 7d arm (*n* = 16), 82.3% in PQ 7.0 mg/kg over 14d arm (*n* = 28), and 79.4% in PQ 7.0 mg/kg over 7d arm (*n* = 27) (log-rank; *p* = 0.0065).

**Conclusions:**

High-dose PQ regimens (7.0 mg/kg total) were safe, well tolerated, and significantly reduced *P. vivax* recurrence in children without G6PD deficiency. Both 7- and 14-day schedules showed comparable efficacy, with rare SAEs and normalization of Hb and methaemoglobinaemia by day 28. Given their similar efficacy, the shorter regimen may offer advantages for adherence and programmatic implementation in endemic settings.

*Trial registration* ClinicalTrials.gov, TRN: NCT05044637, Registration Date: 20 August 2021.

**Supplementary Information:**

The online version contains supplementary material available at 10.1186/s12936-025-05686-y.

## Background

*Plasmodium vivax* is the most widely distributed human malaria parasite, mainly outside the African continent [1]. Although it has often been regarded as causing a benign self-limiting infection, the overall burden, economic impact, and severity of disease from *P. vivax* have long been underestimated [2, 3]. In children, the infection not only threatens health and education [4], but also acts as a source of onward parasite transmission, undermining elimination efforts [5–7]. In areas of hyperendemic transmission, it is estimated that *P. vivax* relapses account for 50% to 80% of malaria cases in the paediatric population [5, 6, 8, 9]. Repeated exposure to malaria, whether through relapses or new infections, leads to the gradual development of immunity [10–12]. It has been suggested that both the proportion of relapsing infections and the number of symptomatic relapses decrease with age [6, 8, 9].

For the treatment of *P. vivax* malaria, the World Health Organization (WHO) recommends either chloroquine (CQ) or an artemisinin-based combination therapy (ACT) to treat blood-stage forms, depending on regional drug sensitivities, followed by or alongside a course of primaquine (PQ) or tafenoquine (TQ) to eliminate liver-stage parasites (radical cure) [13]. PQ has remained the primary approved hypnozoiticidal drug for children since 1952; however, most studies on the efficacy of PQ have been conducted in adults, with children comprising only a subset of the recruited population [14, 15]. Given the high burden of disease in young children and the existing challenges with prescribing PQ, efficacy data in this population are essential [15]. Even with the recent recommendation by the WHO for a single-dose TQ to prevent relapses in individuals aged ≥ 16 years, successful implementation will depend on the availability of affordable and accurate point-of-care (POC) quantitative glucose-6-phosphate dehydrogenase (G6PD) testing**,** which is recommended before administering both PQ or TQ to ensure patient safety and guide appropriate use [16, 17].

The primary determinant of PQ efficacy seems to be the total dose administered, rather than the daily dosing schedule [18]. Although the 14-day regimen of 0.25 mg/kg PQ daily (total 3.5 mg/kg) has acceptable efficacy in South America and attenuates the risk of haemolysis in individuals with G6PD deficiency, it fails to prevent relapses everywhere [19–21].^.^Unsupervised 14-day PQ regimens are associated with reduced adherence and effectiveness, while dose supervision becomes impractical [19, 22, 23].

Shorter courses of PQ with higher daily doses do not compromise efficacy and may improve adherence, thus enhancing effectiveness [24]. The WHO guidelines now recommend the high-dose regimen (7.0 mg/kg) of 0.5 mg/kg/day over 14 days or 1.0 mg/kg/day over 7 days [13]. Clinical trials have demonstrated greater effectiveness in preventing recurrences in adults, especially when dosing is supervised, with an acceptable safety profile [19]. However, despite this WHO recommendation, South American countries have not yet incorporated the high-dose regimen into national malaria treatment guidelines. The Brazilian Ministry of Health continues to recommend the standard low-dose regimen 0.5 mg/kg/day for 7 days (3.5 mg/kg total dose) due to: limited access to point-of-care G6PD testing; insufficient safety data in young children; and operational challenges related to adherence in remote areas of the Amazon [25]. There is an urgent need for a more extensive evaluation of PQ’s safety and tolerability in young children in Latin America. Therefore, this trial assessed the safety, tolerability, and efficacy of CQ combined with different PQ regimens: low total dose (3.5 mg/kg total or 0.5 mg/kg/day, over 7 days) and high total dose (7.0 mg/kg total or 1.0 mg/kg/day, over 7 days; or 0.5 mg/kg/day, over 14 days) for the treatment of *P. vivax* malaria.

## Methods

### Study design

CHILDPRIM was an open-label, parallel-group, non-inferiority, randomized controlled trial of children with uncomplicated malaria vivax and no G6PD deficiency (G6PDd), conducted between August 2021 and January 2025 in the Brazilian Amazon. The goal was to study safety, tolerability, and parasitological response among short and long courses of different PQ doses. This trial was conducted at *Fundação de Medicina Tropical Dr. Heitor Vieira Dourado*, a tertiary health unit in Manaus, Amazonas state, and at *Centro de Saúde Agricultor* and *Centro de Saúde Jesuíno Lins*, two primary healthcare facilities in Cruzeiro do Sul, Acre state. Low and high malaria transmission profiles are observed in Manaus and Cruzeiro do Sul, respectively.

This protocol was approved by the Brazilian Committee of Ethics in Human Research (50193921.6.1001.0005) and registered in ClinicalTrials.gov (NCT05044637). The trial was reported following the Consolidated Standards of Reporting Trials (CONSORT) guideline and the STARTER Checklist for Antimalarial Therapeutic Efficacy Reporting.

### Participants

Eligible participants were children under 15 years of age, with uncomplicated vivax malaria, who were symptomatic with fever ≥ 37.5 °C at the moment of the visit or with a history of fever in the preceding 48 h, plus a minimum weight of 5 kg, with slide-confirmed *P. vivax* mono-infection, haemoglobin (Hb) > 7.0 g/dL, G6PD activity ≥ 4.0 IU/g Hb and absence of severe malnutrition (as per WHO criteria) [26] or underlying chronic or severe disease according at the discretion of the clinician. Exclusion criteria were mixed infection, pregnancy or lactation, inability to receive oral treatment, blood transfusion within the past 90 days, previous malaria episodes within 30 days, any hypersensitivity to study drugs, or signs of severe malaria.

### Interventions

All participants received CQ for 3 days (10 mg/kg on day 1, and 7.5 mg/kg on days 2 and 3), and PQ starting on the same day, with PQ administered for 7 or 14-days regimens according to random allocation at a 1:1:1 ratio to the following treatment arms: (1) standard-dose PQ (3.5 mg/kg total over 7 days); (2) high-dose long-course PQ (7.0 mg/kg total over 14 days); or (3) high-dose short-course PQ (7.0 mg/kg total over 7 days). The 7-day PQ regimen of 3.5 mg/kg total dose is the current recommended treatment for *P. vivax* in Brazil [25]. In contrast, a 14-day PQ regimen of 7.0 mg/kg total dose is recommended for the treatment of recurrences in Brazil and in cases of resistant *P. vivax* in Southeast Asia [13, 25]. For children unable to swallow, tablets were dissolved in 1 to 5 mL of water and administered as a suspension. PQ 5 mg and 15 mg tablets were obtained from Svizera, Switzerland, and Farmanguinhos, Brazil, respectively. CQ 150 mg tablets were obtained from Farmanguinhos, Fiocruz, Brazil. All drugs are provided free of charge in the public health system after confirmation of malaria by thick blood smear microscopy (Giemsa-stained), as per national guidelines. Quality control of the drugs is routinely performed by INCQS. The number of CQ and PQ pills was calculated using weight bands, as already used by the Brazilian MoH (Supplementary Fig. 1). Pills were given in an envelope with further printed instructions after pharmaceutical counselling. Remaining pills were counted at each visit to estimate compliance (semi-supervised strategy).

### Randomization and masking

Once eligible, participants were randomized to one of the three treatment regimens in blocks of five. The randomization list was computer-generated using the R software and the RandomizeR package. No blinding was performed.

### Procedures

At baseline, demographic data and medical history were obtained, followed by physical examination (weight, height, axillary temperature, baseline methaemoglobin (Masimo Radical-7^®^), respiratory rate, pulse oximetry, and pulse rate). Participants were screened for G6PDd using the Standard G6PD Analyzer (SD Biosensor, Korea) semiquantitative test, according to the manufacturer’s instructions. The entire laboratory team was trained according to the manual of procedures of the study, in both sites.

Participants were followed on an outpatient basis and returned for clinical and laboratory evaluations on days 2, 3, 5, 7, 14, 21, 28, 42, and monthly thereafter until day 180. Thick blood smears (TBS) were prepared for parasite identification and quantification as recommended by the Brazilian Ministry of Health [25]. All TBS were read twice by two distinct experienced microscopists, while a third one reviewed any parasitaemia discrepancies. Blood samples for parasitological evaluation were obtained at screening, at all visits, and at recurrences. Baseline Hb levels were measured by fingerprick (HemoCue®, Hb201^+^ System). Hb was assessed at all visits, while methaemoglobin (MetHb) was evaluated until day 28. Female individuals who had experienced menarche were subjected to pregnancy testing. Participants were instructed to return if they developed a fever, at which time a new TBS and blood sample collection would be performed. A medical history was taken, a symptoms questionnaire collected, and any adverse events (AEs) or serious adverse events (SAEs) recorded. The DAIDS grading table was used [27]. After the first relapse, patients were retreated with an ACT plus PQ in high doses over 14 days. 

### Sample size calculation

The primary aim of this trial was to demonstrate the non-inferiority of a PQ regimen of 7.0 mg/kg total dose to the standard regimen of 3.5 mg/kg total dose in terms of safety. The non-inferiority margin was specified on the absolute risk difference scale for adverse events (AEs). PQ test regimen would be considered non-inferior if the upper bound of the one-sided 95% confidence interval for *(risk_test − risk_control)* was ≤  + 5 percentage points. The control arm was PQ 3.5 mg/kg over 7 days. Sample size was calculated for a three-arm design with equal allocation (1:1:1), powered for the most demanding pairwise comparison against control, assuming AE risks of 0.3% (control), 0.4% (PQ 7.0 mg/kg over 14 d), and 2.1% (PQ 7.0 mg/kg over 7 d) until day 28 [28], alpha 0.05 (one-sided), power 90% (beta 0.10), and 15% loss to follow-up, yielding 50 participants per arm (total = 150).

A second interim analysis was performed after 50% of the enrolled patients completed the 180-day follow-up (as per protocol), which revealed a clinically significant increase in recurrence rates in the 3.5 mg/kg over 7 d arm compared to the other treatment groups. Hence, the DSMB recommended to discontinue recruitment early, as continuing the study with the 3.5 mg/kg regimen could expose participants to potentially less effective treatment without added safety benefits, in the context of a clinical trial, even though this was the standard of care arm in Brazil.

### Outcomes

The primary outcomes were the proportion of participants experiencing AEs of any intensity and the proportion of failures until day 180 in the low-dose versus high-dose groups. Secondary endpoints included the evolution of Hb values over time in each study arm and the proportion of AEs in each arm, recurrence-free time, and recurrence frequency.

### Statistical analysis

Data were summarized as frequencies and percentages, means and standard deviations, or medians and interquartile ranges (IQRs), as appropriate. Data are two-sample comparisons for normally distributed variables, which were performed using the Student’s t-test, and for non-normally distributed variables, using the Mann–Whitney U test. Kruskal–Wallis, followed by nonparametric multiple comparison tests, was used to verify differences between arms. Wilcoxon rank-sum test was used to compare continuous variables between two independent groups. To estimate the time to recurrence and the duration participants remained recurrence-free, the Kaplan–Meier method was applied. Participants without recurrence at the end of follow-up were censored at their last recorded visit. The resulting survival curves describe the probability of remaining recurrence-free as a function of time. Unless otherwise stated, all *p*-values are two-tailed, with *p* < 0.05 taken as significant. Statistical analysis was performed using R (version 4.2) and RStudio (RStudio Team, 2022).

## Results

### Population characteristics

**Fig. 1:**
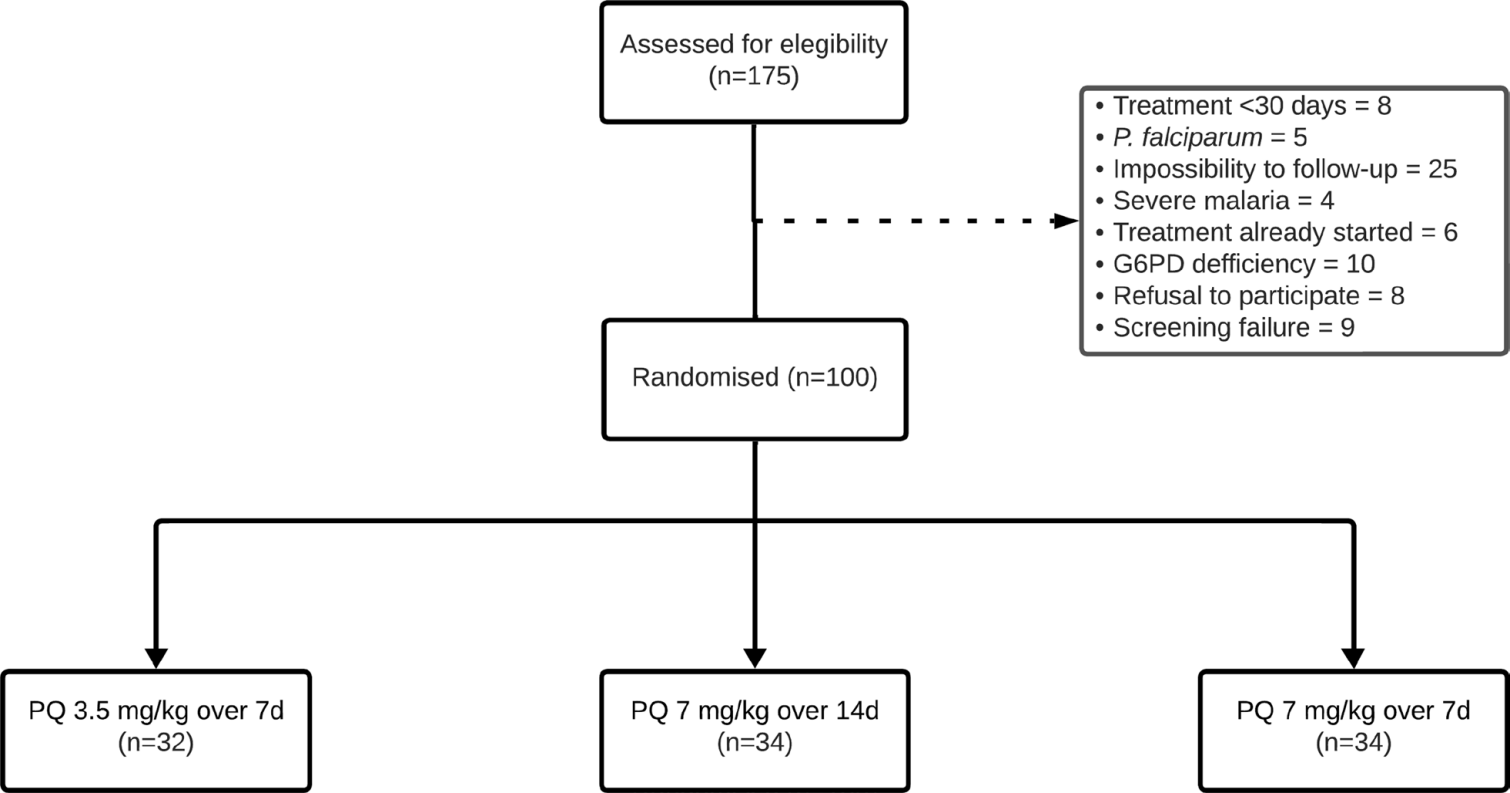
Study flowchart

Among 175 participants screened, 100 were randomized: 32 in the PQ 3.5 mg/kg over 7 d arm, 34 in the PQ 7.0 mg/kg over 14 d arm, and 34 in the PQ 7.0 mg/kg over 7 d arm (Fig. 1). Most participants were recruited in Manaus (72%). Baseline data are summarized in Table 1. Eight children had intermediate G6PD activity, with 8 participants exhibiting 4.0–5.2 IU/g Hb (30–60% activity) (Table 2). 1

**Table 1:** Baseline demographic and clinical characteristics

Variable	Total	PQ 3.5 mg/kg	PQ 7.0 mg/kg	PQ 7.0 mg/kg
	n = 100	over 7d	over 14d	over 7d
		n = 32	n = 34	n = 34
*Gender*^*1*^				
Male	55 (55.0%)	16 (50.0%)	20 (58.8%)	19 (55.9%)
Age (years)^2^	10.1 (6.0–12.0)	11.0 (6.8–12.0)	10.0 (7.3- 12.0)	9.5 (6.0 −12.0)
< 5 years	14 (14.0%)	4 (12.5%)	4 (11.8%)	6 (17.6%)
5–10 years	27 (27.0%)	8 (25.0%)	8 (23.5%)	11 (32.4%)
> 10 years	59 (59.0%)	20 (62.5%)	22 (64.7%)	17 (50.0%)
Previous malaria^1^	46 (46.0%)	13 (40.6%)	10 (29.4%)	16 (47.1%)
Parasite density^2^	4,835 (555–11,310)	3,900 (563–6,064)	6,420 (2,985–10,924)	4,170 (2,306–6,859)
*Clinical characteristic*^*2*^				
Pulse rate (bpm)	114 (100–126)	115 (102–126)	115 (100–126)	113 (100–126)
Respiratory rate (bpm)	21 (18.0–22.0)	21 (18.0–22.0)	22 (19.0–22.0)	21 (18.0- 22.0)
Oximetry (%)	99 (98.0–99.0)	99 (98.0–99.0)	99 (98.0–99.0)	99 (98.0–99.0)
Haemoglobin (g/dL)	12.8 (10.6–15.1)	13.3 (11.4–14.9)	12.4 (10.5–14.3)	12.7 (11.3–13.6)
Methaemoglobin (Sp%)	1.2 (0.3–1.8)	1.4 (0.4, 1.8)	1.5 (0.6–1.7)	1.5 (1.1–1.8)
*Nutritional status*^*3*^				
Undernutrition	4 (4.0%)	1 (3.1%)	1 (2.9%)	2 (5.8%)
Eutrophy	66 (66.0%)	24 (75%)	24 (70.5%)	18 (52.9%)
Overweight	19 (19.0%)	5 (15.6%)	5 (14.7%)	9 (26.4%)
Obesity	11 (11.0%)	2 (6.2%)	4 (11.7%)	5 (14.7%)
*Symptoms at admission*^*1*^				
Fever	100 (100%)	29 (90.6%)	32 (94.1%)	33 (97.1%)
Headache	82 (82.0%)	27 (84.4%)	28 (82.4%)	27 (79.4%)
Chills	81(81.0%)	26 (81.3%)	29 (85.3%)	26 (76.5%)
Vomiting	58 (58.0%)	13 (40.6%)	23 (67.7%)	22 (64.7%)
Loss of appetite	57 (57.0%)	20 (62.5%)	20 (58.8%)	17 (50.0%)
Weakness/asthaenia	55(55.0%)	19 (59.4%)	18 (52.9%)	18 (52.9%)
Dizziness	39 (39.0%)	12 (37.5%)	14 (41.5%)	13 (38.2%)
Sudoresis	38 (38.0%)	13 (40.6%)	11 (32.4%)	14 (41.2%)
Abdominal pain	36 (36.0%)	9 (28.1%)	17 (50.0%)	10 (29.4%)
Dark urine	36 (36.0%)	13 (40.6%)	9 (26.5%)	14 (41.2%)
Malaise	35 (35.0%)	11 (34.4%)	14 (41.2%)	10 (29.4%)
Muscle pain	30 (30.0%)	9 (28.1%)	13 (38.2%)	8 (23.5%)
Nausea	26 (26.0%)	11 (18.3%)	12 (18.2%)	3 (4.9%)

^1^n (%)

^2^Median (IQR)

^3^WHO criteria [26]

**Table 2:** Baseline intermediate G6PD and clinical repercussions

Site	G6PD activity	PQ treatment received	Age	Gender	Parasite density/µL	Baseline Hb	Lowest Hb
*Manaus*	4.3	3.5 mg/kg over 7d	11	F	4,860	11.4	9.8
*C. Sul*	4.5	3.5 mg/kg over 7d	12	F	5,940	14.9	11.0
*C. Sul*	4.5	3.5 mg/kg over 7d	12	M	6,270	11.4	9.5
*C. Sul*	4.7	7.0 mg/kg over 14d	10	M	3,990	11.0	9.4
*C. Sul*	4.7	7.0 mg/kg over 14d	14	M	12,240	14.6	11.2
*C. Sul*	4.2	7.0 mg/kg over 14d	9	M	1,212	14.4	10.0
*C. Sul*	4.9	7.0 mg/kg over 14d	13	F	6,540	15.8	10.0
*C. Sul*	5.2	7.0 mg/kg over 7d	14	F	480	11.4	11.4

Supplementary Table 1 summarizes the median dosing of CQ and PQ used.

### Safety and tolerability

During the 180-day follow-up, a total of 486 AEs were reported for every patient, regardless of the association with the drugs of the study; the PQ 7.0 mg/kg over 14 d arm had the highest number of AEs (*n* = 186), followed by PQ 7.0 mg/kg over 7 d (*n* = 166) and PQ 3.5 mg/kg over 7 d (*n* = 134), *p* = 0.1 (Supplementary Table 2). Among them, 20 were classified as grade 3 and 4 AEs (Table 3). Of these, 16 were considered clinically insignificant, while four required medical evaluation and intervention. These included: one episode of abdominal pain in the PQ 3.5 mg/kg over 7 d arm; one case of methaemoglobinaemia associated with intractable vomiting that necessitated ICU admission due to inability to maintain oral hydration; one case of anaemia requiring blood transfusion in the PQ 7.0 mg/kg over 14 d arm; and a humerus fracture resulting from a bicycle fall (between days 60 and 90 of follow-up), in the PQ 7.0 mg/kg over 7 d arm. All SAEs related to the study interventions occurred within the first seven days after admission. All nine children in whom anaemia (haemoglobin < 8.5 g/dL) was detected during the follow-up are described in Supplementary Table 3, as well as the eight children in whom methaemoglobinaemia grade ≥ 3 was measured (Supplementary Table 4).

**Table 3:** Grade 3 and 4 adverse events during study interventions

Adverse event	Treatment arm			
	PQ 3.5 mg/kg over 7d n = 2^1^	PQ 7.0 mg/kg over 14d n = 10^1^	PQ 7.0 mg/kg over 7d n = 8^1^	*p*^2^
Methaemoglobinaemia	1	3	4	0.6
Anaemia	0	6	3	0.6
Abdominal pain	1	0	0	0.1
Vomiting	0	1	0	> 0.9
Fracture of humerus	0	0	1	> 0.9

^*^ Serious adverse event: 1 abdominal pain, 1 methaemoglobinaemia, 1 anaemia requiring blood transfusion, 1 vomiting

^1^n/N (%)

^2^Fisher's exact test

Of the 486 adverse events (AEs) reported, 398 (81.9%) occurred within the first 28 days after treatment initiation. The most frequent clinical AEs were gastrointestinal symptoms, headache, dizziness, asthenia/weakness, and chills, with a similar distribution across treatment arms (Table 4). Acute vomiting episodes within 1 h of drug administration occurred in all treatment arms on days 0 to 2; all affected participants received a replacement dose according to protocol, and none required treatment discontinuation due to drug intolerance.

**Table 4:** Frequencies of adverse events associated with study interventions within the first 28 days after inclusion

		Treatment arm	
Adverse event	PQ 3.5 mg/kg over 7 d, n = 66	PQ 7.0 mg/kg over 14 d, n = 82	PQ 7.0 mg/kg over 7 d, n = 85
GI symptoms	43 (66.6%)	58 (70.7%)	63 (74.1%)
Abdominal pain	11 (16.6%)	18 (21.9%)	15 (17.6%)
Vomiting	8 (12.1%)	18 (21.9%)	17 (20.0%)
Diarrhoea	9 (13.6%)	8 (9.7%)	11 (12.9%)
Nausea	6 (9.0%)	4 (4.8%)	7 (8.2%)
Decreased appetite	4 (6.0%)	5 (6.0%)	8 (9.4%)
Vomiting of medication*	3 (4.5%)	2 (2.4%)	4 (4.7%)
Dehydration	2 (3.0%)	3 (3.6%)	1 (1.1%)
Headache	9 (13.6%)	10 (12.1%)	8 (9.4%)
Dizziness	6 (9.0%)	3 (3.6%)	1 (1.1%)
Asthaenia/weakness	4 (6.0%)	2 (2.4%)	3 (3.5%)
Cyanosis	2 (3.0%)	2 (2.4%)	4 (4.7%)
Malaise	1 (1.5%)	4 (4.8%)	3 (3.5%)
Chills	1 (1.5%)	3 (3.6%)	3 (3.5%)

^*^ Within 1 h of drug administration

Anaemia was reported in 73 participants, with similar frequencies in the PQ 3.5 mg/kg over 7-day 15.1% (17/112) and PQ 7.0 mg/kg over 7-day 15.3% (21/137) arms, and a slightly higher proportion in the PQ 7.0 mg/kg over 14-day group 23.4% (35/149).

Methaemoglobinemia was identified in 92 participants, with 25.8% (29/112) in the 3.5 mg/kg over 7-day group, 21.4% (32/149) in the 7.0 mg/kg over 14-day group, and 22.6% (31/137) in the 7.0 mg/kg over 7-day group.

**Fig. 2:**
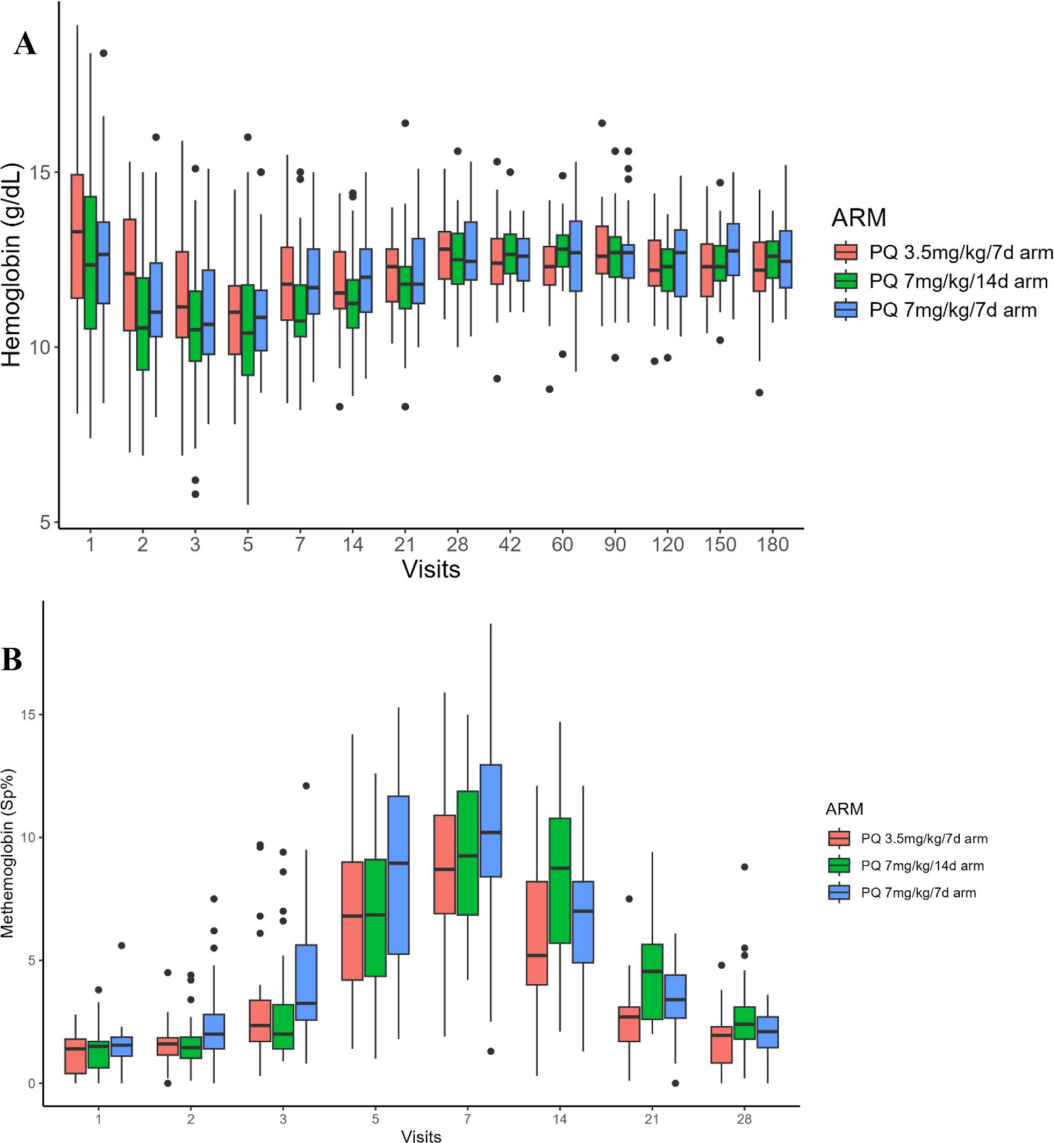
Haemoglobin (**A**) and methaemoglobinaemia (**B**) levels of participants throughout the follow-up period

At baseline, haemoglobin and methaemoglobin concentrations did not differ among groups (Fig. 2). The Hb concentrations dropped during PQ therapy in all groups, with a median (IQR) decline to a nadir of 2.3 (−3.1, −0.8) g/dL, 1.9 (−3.0, 0.0) g/dL, and 1.6 (−2.5,−0.9) g/dL for PQ 3.5 mg/kg over 7 d arm, PQ 7.0 mg/kg over 14 d arm and PQ 7.0 mg/kg over 7 d, respectively (*p* = 0.6). The median Hb levels had returned to baseline by day 28 in all treatment groups (Fig. 2A).

All arms showed elevations in MetHb, particularly from day 3 to day 14. Peak MetHb concentrations occurred on day 7 with a median level of 7.9 (6.1, 9.7), 7.4 (5.6, 10.3), and 8.8 (6.2, 11.3) from baseline for PQ 3.5 mg/kg over 7 d arm, PQ 7.0 mg/kg over 14 d arm and PQ 7.0 mg/kg over 7 d, respectively (*p* = 0.6). Elevated levels observed early in treatment diminished over time, returning to near baseline by day 28 (Fig. 2B). Owing to methaemoglobinaemia, treatment was discontinued on day 7 for two participants in PQ 7.0 mg/kg over 14 d arm and on day 5 for one participant in PQ 7.0 mg/kg over 7 d arm. One participant also had the medication interrupted on day 7 due to intractable vomiting and methaemoglobinaemia (SAE), and one participant self-interrupted PQ treatment on day 9. Both were allocated to the PQ 7.0 mg/kg over 14 d arm.

### Recurrences

**Fig. 3:**
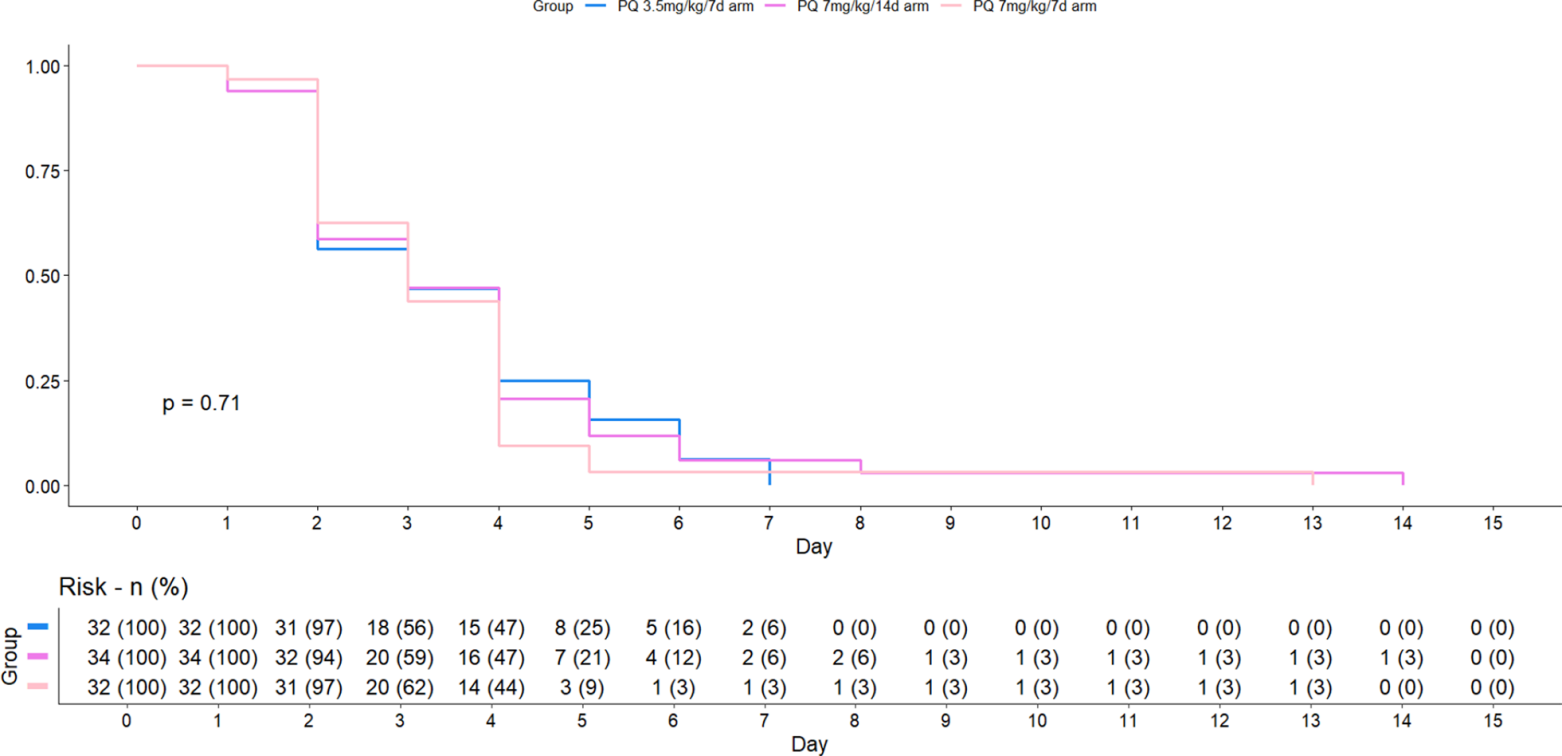
Time to parasite clearance on the thick blood smear

All participants cleared peripheral parasitaemia within 3.4 days (± 2.1) of treatment initiation, with no significant difference between treatment groups (Fig. 3). During follow-up, 30 recurrences were documented across all treatment arms. Out of these, 4 (13.3%) occurred in children under 5 years of age, 12 (40.0%) in those aged 5–10 years, and 14 (46.7%) in participants older than 10 years (Supplementary Table 5).

**Fig. 4:**
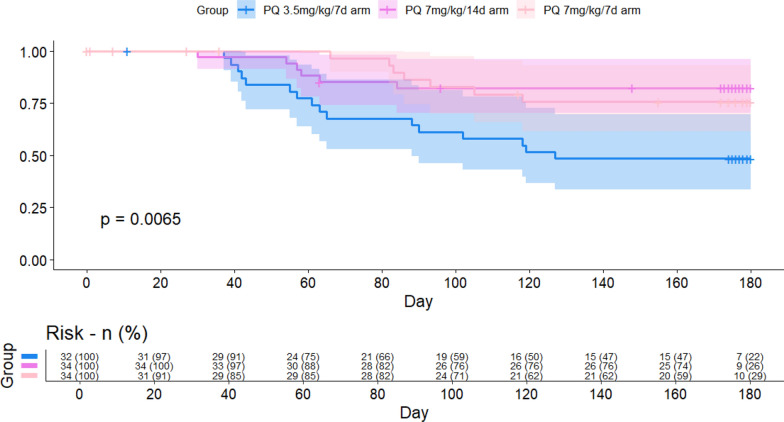
Kaplan–Meier analysis of the *P. vivax* recurrence at 180 days. Vertical notches denote right-censored observations. The shaded areas represent 95% confidence intervals

Kaplan–Meier estimates of the percentage of participants who were free from recurrence at day 180 were 50% in the PQ 3.5 mg/kg over 7 d arm (*n* = 16), 82.3% in PQ 7.0 mg/kg over 14 d arm (*n* = 28), and 79.4% in PQ 7.0 mg/kg over 7 d arm (*n* = 27) (Fig. 4). After controlling for age, baseline parasitaemia, methaemoglobinaemia on day 7, CQ total dose, and PQ total dose, only the PQ total dose was significantly associated with an increased rate of recurrence (*p* = 0.032; Supplementary Table 6).

## Discussion

A short high-dose PQ regimen was likely safe and well-tolerated in this small study with children without G6PDd. Notably, this is the first report on using PQ at this dosage for radical cure in the paediatric population in Latin America, as well as its application in children with intermediate levels of G6PD activity. However, this study focused on efficacy under a controlled scenario, and effectiveness analyses could change the real-life impact of higher doses of PQ over 7 days.

In this study, children with intermediate G6PD activity generally tolerated high-dose PQ regimens without major safety concerns. No clinically significant haemolytic events were observed in most participants within this subgroup. One female participant with G6PD activity of 5.9 IU/gHb required a blood transfusion; however, her clinical picture involved a primary *P. vivax* infection, delayed treatment initiation after more than 10 days of symptoms, high parasite density upon admission, and a low baseline Hb level of 7.2 g/dL. These factors suggest that the adverse outcome cannot be attributed solely to G6PD activity. While these findings support the relative safety of PQ in children with G6PD activity ≥ 4.0 IU/gHb, they reinforce the importance of close clinical monitoring, particularly in heterozygous females and in patients presenting with delayed diagnosis, elevated parasitaemia, or preexisting anaemia.

Despite promising results, safety concerns persist, especially regarding haemolysis in G6PDd children. While weekly PQ regimens have shown a promising safety and effectiveness profile [29, 30], these practicalities underscore the need for refined risk stratification and individualized dosing strategies, particularly in regions with high G6PDd prevalence [31]. Optimal dosing of PQ is crucial to ensure efficacy and safety [18]. Higher total doses are superior to the lower dose in preventing relapses [24], particularly in younger children who have reduced immunity [15, 32, 33]. However, age-based dosing is a significant contributor to underdosing, which leads to higher recurrence rates [34]. Therefore, a shift towards weight band-based dosing regimens is necessary to ensure accurate dosing and better outcomes.

Adherence remains a significant challenge influencing treatment outcomes, especially in the Amazon [35]. Shorter high-dose regimens have shown potential in improving adherence but also pose risks of increased gastrointestinal side effects and haemolysis [18]. While all PQ doses are associated with gastrointestinal symptoms, higher daily doses increased the intensity and frequency of these symptoms [19]. Nevertheless, adherence is generally better in clinical trials, suggesting real-world compliance may vary. Directly observed therapy could be a solution, though its feasibility in resource-limited settings needs further evaluation [35].

Recent studies have demonstrated that the formation of MetHb, a product of Hb oxidation induced by PQ and its reactive metabolites, occurs in a dose-dependent manner [14, 36]. On average, for each 0.1 mg/kg increment in the daily PQ dose, there is an approximate 0.34 percentage point increase in MetHb concentration on day 7 of treatment, with robust statistical significance (*p* < 0.001) [36]. This finding suggests that MetHb may serve as a pharmacodynamic marker of the extent of PQ bioactivation, allowing for indirect inferences about the potential efficacy of treatment.

Interindividual variation in the MetHb response to PQ may also be partially attributed to heterogeneity in CYP2D6 enzyme activity, which is influenced by genetic polymorphisms resulting in ultrarapid, normal, intermediate, or poor metabolizer phenotypes [37]. However, the absence of genotypic or phenotypic data on CYP2D6 activity in this study limits the interpretation of individual variation in MetHb response. This limitation underscores the need for future studies that integrate CYP2D6 genotyping, MetHb quantification, and clinical outcomes in paediatric cohorts to enhance the personalization of primaquine therapy. In addition, the limited data available for children under one year of age highlight the need for focused safety studies in this age group.

The study also identified SAEs occurring more frequently in groups with higher parasite densities at admission, particularly within the first seven days of treatment. This early finding suggests that early treatment days are crucial for monitoring and managing potential adverse effects. Moreover, the precise administration of PQ is essential as factors like vomiting, diarrhoea, pharmaceutical formulation variations, and ethnicity can affect drug absorption and increase the risk of suboptimal dosing [37].

Another significant barrier for radical treatment of *P. vivax* malaria in childhood is the lack of paediatric-friendly formulations, such as dispersible tablets. Developing such formulations is crucial for enhancing dosing accuracy and adherence in children [1]. Besides, the absence of widespread POC G6PD testing remains a challenge. Although weekly PQ regimens offer an alternative in case the G6PD status is unknown [29], broader implementation of reliable testing methods is still crucial for optimizing treatment safety.

This study has several limitations. First, the absence of genotypic assessments of CYP2D6 activity precluded a more comprehensive analysis of the relationship between primaquine metabolism, methaemoglobinaemia, and recurrence. Although ethically justified, the early termination of recruitment based on interim analysis reduced the final sample size, potentially affecting the statistical power to detect differences in secondary outcomes, particularly among children under five or those with intermediate G6PD activity. Additionally, no children under one year of age were included, restricting the applicability of the results to this vulnerable age group. Treatment adherence was monitored through pill counts and caregiver reports; however, not all doses were directly observed, particularly in the 14-day regimen, which may have introduced variability in adherence. Most participants were recruited in Manaus, resulting in an imbalance between sites that may have influenced recurrence outcomes given the different transmission intensities. There is also a potential underreporting of mild and self-limited AEs, especially beyond the first month of follow-up. Finally, the absence of pharmacokinetic measurements of PQ and metabolites limited our ability to correlate drug exposure with treatment efficacy and safety outcomes. Both high-dose PQ regimens demonstrated superior efficacy in reducing *P. vivax* recurrence compared to the standard-dose regimen, reinforcing their potential as preferred strategies for radical cure in children without G6PD deficiency. In the clinical trial scenario, the study was halted due to the lower efficacy of the low-dose arm; however, this regimen remains the recommended one in Brazil, which makes the halt questionable. Future studies should assess the comparative cost-effectiveness of the short versus long-course schedules, balancing clinical benefits, adherence, and operational feasibility in endemic settings.

## Conclusions

This study demonstrates that high-dose PQ regimens are safe for the radical cure of *P. vivax* malaria in children without G6PD deficiency in the Brazilian Amazon, significantly reducing recurrence rates compared to the current standard. Both 7- and 14-day schedules were well tolerated, with rare SAEs and normalization of Hb and methaemoglobinaemia by day 28. Children with intermediate G6PD activity also showed good tolerability, though monitoring remains crucial. Given their comparable efficacy, the shorter 7-day high-dose regimen may offer advantages in terms of adherence and operational feasibility, and thus could be prioritized in endemic settings, while further studies should assess its comparative effectiveness. The findings underscore the need for weight-based dosing, improved adherence strategies, paediatric-friendly formulations, and broader G6PD testing to optimize outcomes in endemic settings.

## Supplementary Information

Additional file1

Additional file2

## Data Availability

The data that support the findings of this study are available from the corresponding author upon reasonable request.
