# Supplementary material for: Safety, tolerability, and efficacy of high versus low-dose, short versus long-course daily primaquine for the radical cure of uncomplicated Plasmodium vivax malaria in children under 15 years of age: an open-label, non-inferiority, randomized controlled trial (CHILDPRIM)
Source: Malar J. 2025 Dec 22;25:58. doi: 10.1186/s12936-025-05686-y (PMC12836904; doi:10.1186/s12936-025-05686-y)
Supplement: Supplementary file 2 — Additional file2 [file 12936_2025_5686_MOESM2_ESM.docx]

**Supplementary Figures**

1. Treatment schedule by weight band and primaquine regimen in the CHILDPRIM trial.

**
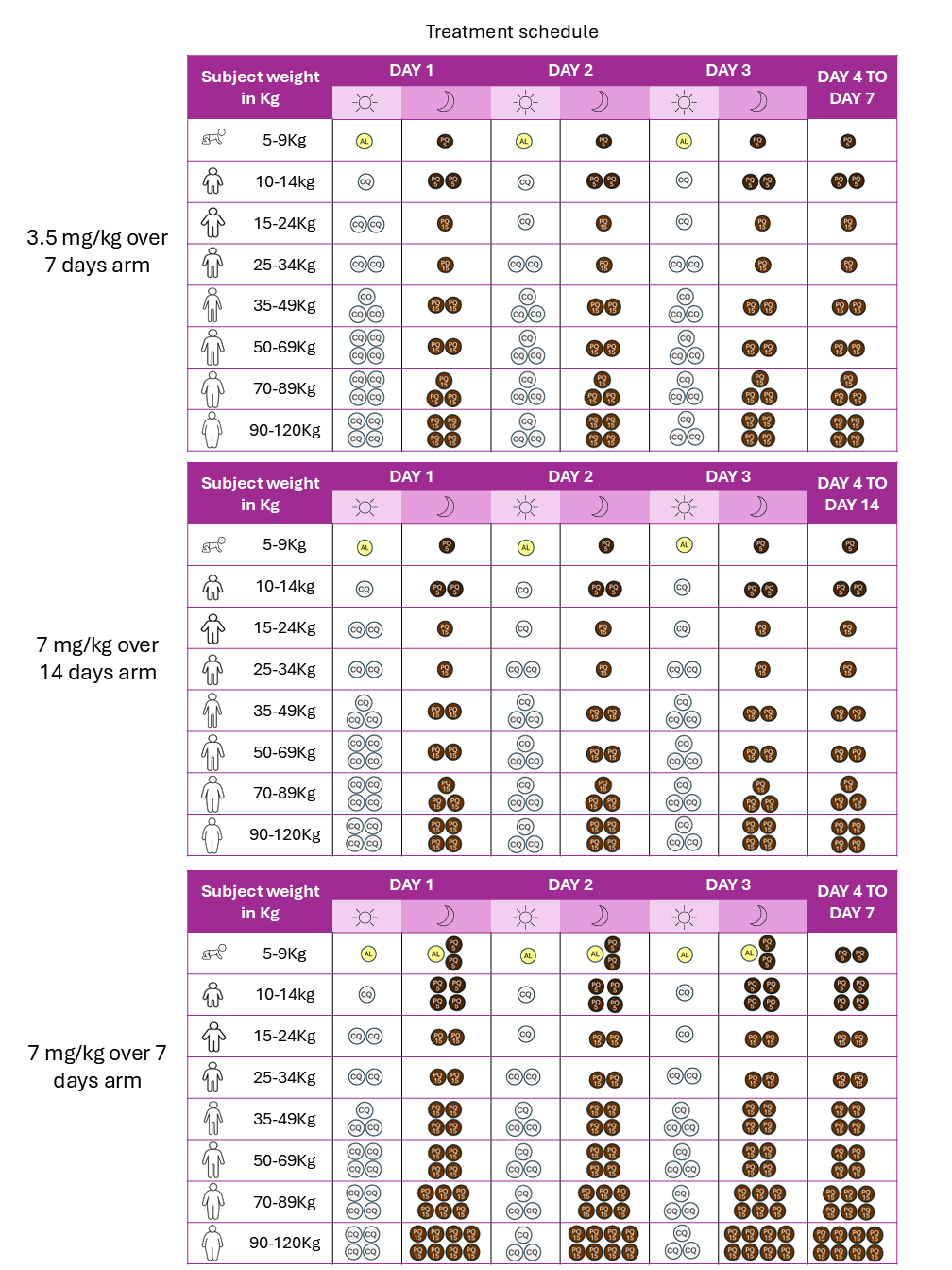
**

.

**Supplementary Tables**

**Supplementary Table 1.** Median CQ and PQ dose per arm.

| Variable | Dose x weight | | | *p^2^* |
| --- | --- | --- | --- | --- |
|  | PQ 3.5 mg/kg over 7d  n=32^1^ | PQ 7.0 mg/kg over 14d  n=34^1^ | PQ 7.0 mg/kg over 7d  n=34^1^ |  |
| PQ (mg/kg/day) | 0.65 (0.52-0.78) | 0.64 (0.52-0.71) | 1.26 (1.04-1.47) | <0.001 |
| PQ (total dose) | 4.56 (3.67-5.44) | 8.94 (7.27-10.02) | 8.78 (7.24-10.27) | <0.001 |
| CQ (mg/kg) | 30.30 (28.53-34.83) | 29.35 (27.37-33.14) | 29.62 (27.05-33.21) | 0.57 |
| *^1^* Median (IQR) | | | | |
| *^2^* Kruskal-Wallis rank sum-test | | | | |

**Supplementary Table 2.** Adverse events per arm (frequency and intensity).

| AE | Treatment arm | | | *p^2^* |
| --- | --- | --- | --- | --- |
|  | PQ 3.5 mg/kg over 7d  n=134*^1^* | PQ 7.0 mg/kg over 14d  n=186*^1^* | PQ 7.0 mg/kg over 7d,  n=166*^1^* |  |
| Event intensity |  |  |  | 0.1 |
| Grade 1 | 86 (64.0%) | 93 (50.0%) | 90 (54.0%) |  |
| Grade 2 | 46 (34.0%) | 82 (44.0%) | 68 (41.0%) |  |
| Grade 3 | 2 (1.5%) | 9 (4.7%) | 8 (4.8%) |  |
| Life-threatening | 0 (0%) | 1 (0.5%) | 0 (0%) |  |
| *^1^* n (%) | | | | |
| *^2^* Fisher’s exact test | | | | |

**Supplementary Table 3.** Characteristics and outcomes of participants with grade ≥3 anaemia across high-dose primaquine regimens

| Participant  ID | Treatment arm | Age (years) | Sex | G6PD activity (IU/g Hb) | | G6PD activity (IU/g Hb)***** | | Parasite density (μL⁻¹) D1 | | Baseline Hb (g/dL) | | Day of Hb nadir | | Hb nadir (g/dL) | Clinical action | | Outcome /  Hb D28 (g/dL) | |  |
| --- | --- | --- | --- | --- | --- | --- | --- | --- | --- | --- | --- | --- | --- | --- | --- | --- | --- | --- | --- |
| P6 | PQ 7.0 mg/kg/14d | 8 | M | 11.3 | 11.5 | | 7,860 | | 10.5 | | 2 | | 8.0 | | | Supportive care only | | Full recovery, Hb 11.5 | |
| P7 | PQ 7.0 mg/kg/14d | 11 | M | 9.0 | 9.0 | | 630 | | 9.6 | | 3 | | 8.2 | | | Supportive care only | | Full recovery, Hb 12.4 | |
| P9 | PQ 7.0 mg/kg/7d | 11 | M | 8.7 | 8.7 | | 3,360 | | 10.6 | | 3 | | 8.0 | | | Supportive care only | | Full recovery, Hb 12.1 | |
| P30 | PQ 7.0 mg/kg/14d | 4 | M | 7.4 | ---- | | 12,240 | | 13.0 | | 3 | | 6.2 | | | Supportive care only | | Full recovery, Hb 11.5 | |
| P46 | PQ 7.0 mg/kg/14d | 2 | M | 14.2 | 14.0 | | 11,280 | | 11.4 | | 5 | | 7.9 | | | Supportive care only | | Full recovery, Hb 11.8 | |
| P65 | PQ 7.0 mg/kg/14d | 5 | F | 6.8 | 7.1 | | 11,610 | | 9.2 | | 5 | | 7.0 | | | Supportive care only | | Full recovery, Hb 13.1 | |
| P66 | PQ 7.0 mg/kg/14d | 12 | F | 5.9 | 5.9 | | 9,030 | | 7.4 | | 5 | | 5.5 | | | Transfusion (1 unit RBC) | | Full recovery, Hb 13.5 | |
| P88 | PQ 7.0 mg/kg/7d | 2 | F | 11.0 | 11.1 | | 36,600 | | 9.7 | | 3 | | 7.8 | | | Supportive care only | | Full recovery, Hb 11.8 | |
| P96 | PQ 7.0 mg/kg/7d | 6 | M | 9.6 | 9.8 | | 5,130 | | 9.6 | | 2 | | 8.1 | | | Supportive care only | | Full recovery, Hb 10.3 | |

* G6PD testing was repeated in participants with grade ≥3 anaemia

Parasite density is expressed as asexual parasites per µL (assuming 6000 WBC/µL). Supportive care only = clinical monitoring and symptomatic management without specific intervention

**Supplementary Table 4.** Characteristics and clinical outcomes of participants who developed methemoglobinemia grade ≥3.

| Participant ID | Treatment arm | Sex | Age (years) | G6PD activity (IU/g Hb) | Baseline MetHb (%) | Highest MetHb (%) | Day of Highest MetHb (%) | Symptoms / Clinical Notes | Clinical Management |
| --- | --- | --- | --- | --- | --- | --- | --- | --- | --- |
| P1 | PQ 7.0 mg/kg/7 days | M | 13 | 6.8 | 0.5 | 18.7 | D7 | Abdominal pain, cyanosis and pallor. | Participant had completed the last dose of treatment. Close monitoring, supportive care only. |
| P28 | PQ 7.0 mg/kg/7 days | M | 10 | 6.0 | 0.5 | 15.3 | D7 | Headache, cyanosis and pallor. | Participant had completed the last dose of treatment. Close monitoring, supportive care only. |
| P62 | PQ 7.0 mg/kg/14days | F | 8 | 8.3 | 1.7 | 15.0 | D7 | Abdominal pain, headache, nausea, vomiting and cyanosis | Primaquine interrupted. Close monitoring, supportive care only. |
| P68 | PQ 3.5 mg/kg/7 days | M | 14 | 8.7 | 2.8 | 15.9 | D7 | Asthenia, nausea and cyanosis. | Participant had completed the last dose of treatment. Close monitoring, supportive care only. |
| P74 | PQ 7.0 mg/kg/14 days | M | 10 | 7.3 | 1.5 | 13.6 | D7 | Abdominal pain, loss of appetite, malaise, cyanosis and pallor. | Primaquine interrupted. Close monitoring, supportive care only. |
| P75 | PQ 7.0 mg/kg/14 days | F | 11 | 8.1 | 1.7 | 14.0 | D7 | Cyanosis, pallor intractable vomiting and dehydration | Primaquine interrupted, ICU + supplementary oxygen |
| P79 | PQ 7.0 mg/kg/7days | M | 13 | 6.3 | 1.7 | 15.3 | D7 | Abdominal pain, headache, pallor and cyanosis | Participant had completed the last dose of treatment. Close monitoring, supportive care only. |
| P92 | PQ 7.0 mg/kg/7 days | F | 3 | 7.4 | 1.3 | 15.3 | D5 | Asthenia, headache dizziness, cyanosis and pallor. | Primaquine interrupted Close monitoring, supportive care only. |

**Supplementary Table 5.** Recurrence per age and arm.

|  | PQ 3.5mg/kg over 7d | | | PQ 7.0 mg/kg over 14d | | | PQ 7.0 mg/kg over 7d | | |
| --- | --- | --- | --- | --- | --- | --- | --- | --- | --- |
|  | No Recurrence, n=17^1^ | Recurrence, n=15*^1^* | p*^2^* | No Recurrence, n=26*^1^* | Recurrence, n=8*^1^* | p*^2^* | No Recurrence, n=27*^1^* | Recurrence  n=7*^1^* | p*^2^* |
| Age (y) |  |  | 0.4 |  |  | >0.9 |  |  | 0.3 |
| < 5 | 3 (17.6%) | 1 (6.3%) |  | 3 (11.5%) | 1 (12.5%) |  | 4 (14.8%) | 2 (28.6%) |  |
| 5-10 | 3 (17.6%) | 6 (40.0%) |  | 11 (42.3%) | 3 (37.5%) |  | 11 (40.7%) | 3 (42.9%) |  |
| > 10 | 11 (64.7%) | 8 (53.3%) |  | 12 (42.6%) | 4 (50.0%) |  | 12 (44.4%) | 2 (28.6%) |  |
| *^1^* n (%) | | | | | | | | | |
| *^2^* Fisher’s exact test | | | | | | | | | |

**Supplementary Table 6.** Assessment of potential predictors of *P. vivax* recurrence: comparison between recurrence and non-recurrence groups

| Variable | Recurrence | | *p^2^* |
| --- | --- | --- | --- |
|  | No, n=71^1^ | Yes, n=29^1^ |  |
| Parasitaemia - D1 | 4,185 (1,485, 7,793) | 5,115 (1,095, 7,050) | >0.9 |
| PQ (mg/kg/day) | 0.4 (0.3, 0.5) | 0.4 (0.3, 0.5) | 0.3 |
| PQ (total dose) | 7.6 (6.3, 9.7) | 6.3 (4.6, 8.8) | 0.032 |
| CQ (mg/kg) | 30.3 (26.5, 34.1) | 29.0 (27.1, 31.9) | 0.3 |
| MetHb | 9.3 (6.9, 11.9) | 9.6 (7.4, 11.8) | 0.6 |
| *^1^* Median (IQR) | | | |
| *^2^* Wilcoxon rank sum test | | | |
